# Repurposing the Fibrosis-4 Score in Rheumatoid Arthritis: Data from the ESPOIR Cohort

**DOI:** 10.3390/jcm13071905

**Published:** 2024-03-26

**Authors:** Renaud Felten, Thibaut Fabacher, Nathanaël Sedmak, Jean Sibilia, Christelle Sordet, Emmanuel Chatelus, Francis Berenbaum, Bernard Combe, Adeline Ruyssen-Witrand, Olivier Vittecoq, Nicolas Meyer, Jacques-Eric Gottenberg

**Affiliations:** 1 National Reference Center for Rare Auto-Immune Diseasesest Sud-Ouest (RESO), Department of Rheumatology, Hôpitaux Universitaires de Strasbourg, 67091 Strasbourg, France; renaud.felten@chru-strasbourg.fr (R.F.);; 2 Department of Public Health, Hôpitaux Universitaires de Strasbourg, 67091 Strasbourg, Francenicolas.meyer@chru-strasbourg.fr (N.M.); 3 Department of Rheumatology, INSERM, AP-HP Saint-Antoine Hospital, Sorbonne University, 75005 Paris, France; 4 Faculty of Medicine, Montpellier University, 34090 Montpellier, France; 5 Department of Rheumatology, Toulouse University Toulouse III Paul Sabatier, 31400 Toulouse, France; 6 Department of Rheumatology & CIC-CRB1404, Rouen University Hospital, Normandie University, UNIROUEN, 76000 Rouen, France

**Keywords:** rheumatoid arthritis, FIB-4, prognostic factor, comorbidities

## Abstract

**Background:** We aimed to evaluate the value of the Fibrosis-4 (FIB-4) score as a prognostic factor in RA in the prospective ESPOIR cohort. **Methods:** We included patients from the ESPOIR cohort with a diagnosis of RA according to ACR/EULAR criteria. The formula for the FIB-4 score is as follows: [age (years) × aspartate transaminase level (U/L)]/[platelet count (10^9^/L) × alanine aminotransferase level (U/L)^1/2^]. We used a linear mixed-effects model with a random effect of patient to account for repeated measures over time. **Results:** Overall, 647 of the 813 patients included met the ACR/EULAR criteria for RA, with no differential diagnosis during the first 10 years of follow-up. Of these patients, at baseline, 633 had a calculable FIB-4 score. Median FIB-4 score was 0.75 (interquartile range 0.53–0.99). On multivariate analysis, FIB-4 score was not independently associated with progression of Disease Activity Score in 28 joints over 10 years of follow-up, unlike baseline C-reactive protein level and SJC. Baseline FIB-4 score was not associated with the modified Sharp score at 5-year follow-up, unlike age and ACPAs. FIB-4 score was not associated with mortality (hazard ratio 1.1 [95% CI 0.46; 2.8], *p* = 0.77) or major adverse cardiovascular events (0.46 [0.13; 1.6], *p* = 0.22) over the 10-year follow-up. No significant change in FIB-4 score over time was related to treatments. **Conclusions:** The present prospective cohort study did not find a prognostic role of FIB-4 score in RA. Reassuringly, FIB-4 score was not increased with DMARD treatment after 10 years of follow-up.

## 1. Introduction

Rheumatoid arthritis (RA), a chronic autoimmune disease, not only imposes challenges on the joints but also intricately affects systemic health. The liver, although traditionally considered uninvolved in the primary pathology of RA, plays a role in the holistic management of this condition. Disturbed levels of liver enzymes are common in patients with RA (20–50% of patients) and transaminase monitoring is part of the biological follow-up of most RA patients. Monitoring liver health, particularly through the Fibrosis-4 (FIB-4) score, has garnered increasing attention as a potentially important facet of RA care. The Fibrosis-4 (FIB-4) score [1] is easily calculated using the patient’s age, platelet count, and aspartate aminotransferase (AST) and alanine aminotransferase (ALT) levels. The FIB-4 score is already used by gastroenterologists to non-invasively screen for liver fibrosis.

In this context, we delve into the novel prospect of utilizing the FIB-4 score as a predictive tool for the course of RA, shedding light on its potential implications for patient management and overall care strategies.

Non-alcoholic fatty liver disease (NAFLD) covers a range of liver issues, spanning from basic fatty liver (steatosis) to non-alcoholic steatohepatitis (NASH). The latter is linked to an increased risk of advancing to conditions such as fibrosis, cirrhosis, and hepatocellular carcinoma. The average prevalence of NAFLD in the world population is estimated at 25.2%. Approximately 15% to 20% of people with NAFLD will have NASH. Assessment of liver fibrosis is critical to the management of these patients. The FIB-4 score can correctly identify patients with severe fibrosis (F3–F4). An FIB-4 score ≤1.3 has a negative predictive value of 90% and >2.67 a positive predictive value of 80% in patients with NASH [2].

Although rheumatoid arthritis (RA) can occur across various age groups, its occurrence becomes more prevalent with advancing age [3]. Crowson et al. [4] demonstrated that the cumulative lifetime risk of RA is less than 1% before the age of 50, but significantly escalates for both genders around the age of 60, reaching a plateau after 80 years [5]. The trajectory of the disease remains uncertain concerning whether the disease course is more favorable or unfavorable in older individuals with RA compared with their younger counterparts, and the existing data present conflicting viewpoints [5].

Platelets have been shown to have important immune effector functions in RA [6]. Activated platelets generate pro-inflammatory microparticles found in both the bloodstream and the synovium. These microparticles play a role in the systemic inflammatory processes associated with RA. Thus, platelets could be seen as relevant biomarkers to reflect both disease activity and response to treatment [7,8]. Platelet count as well as liver enzyme assessment are performed on a regular basis in RA patients receiving disease-modifying antirheumatic drugs (DMARDs), which usually require laboratory monitoring. Liver enzymes are interesting dysmetabolic markers in terms of management of comorbidities promoted by EULAR [9]. Therefore, combining patient age, platelet count, and transaminase levels using the FIB-4 score could be of interest in RA in terms of this management of comorbidities. In the same way as is done for repurposing of drugs for another indication, scores used in some disciplines could be repurposed for other indications [10].

In a cohort of 978 patients who had received methotrexate (MTX) for RA, 4.7% exhibited MTX-associated NAFLD with elevated serum enzyme levels [11]. A Korean retrospective single-center study showed that FIB-4 score at diagnosis could predict mortality (from any cause) in RA patients [12]. The FIB-4 score has never been evaluated in a prospective follow-up of patients with recent RA.

In this study, we used the Evaluation et Suivi de Polyarthrites Indifférenciées Récentes (ESPOIR) cohort, a longitudinal, prospective initiative designed to unravel the complexities of early RA. The ESPOIR cohort, which includes adults aged between 18 and 70 with possible early-onset RA, has provided a unique insight into the developmental trajectory of RA and its multifaceted impacts. By analyzing data from this large cohort, we aim to uncover new insights into the prognostic value of the Fibrosis-4 (FIB-4) score, a measure that goes beyond joints to encompass broader systemic implications in the context of RA. The primary objective of our study was to evaluate the prognostic value of baseline FIB-4 score for the course of RA.

## 2. Materials and Methods

### 2.1. Patients and Main Study Outcomes

Patients included in this study were part of the ESPOIR cohort, ensuring a focused analysis on early RA cases without prior exposure to disease-modifying antirheumatic drugs (DMARDs). The selection criteria aimed to capture a homogenous group for a more nuanced exploration of the FIB-4 score’s prognostic value in RA patients. ESPOIR is a longitudinal prospective cohort of adults (18–70 years old) with possible early RA (ClinicalTrials.gov: NCT03666091) [13]. We included patients from the ESPOIR cohort with a diagnosis of RA according to ACR/EULAR criteria without an established differential diagnosis during the first 10 years of follow-up.

The main study outcomes included demographical characteristics (age, sex); RA disease activity (Disease Activity Score in 28 joints—erythrocyte sedimentation rate [DAS28-ESR], number of tender and swollen joints), C-reactive protein (CRP) level, ESR, structural damage evaluated by the van der Heijde-modified Sharp score [14], and response to treatments (EULAR response, DAS28-ESR remission [15]).

Mortality and the main cardiovascular comorbidities were studied in terms of the FIB-4 score. Major cardiovascular adverse events (MACEs) were defined as a composite of myocardial infarction, stroke, and hospitalization because of heart failure or revascularization.

The following treatments were studied: use of MTX or not, cumulative dose of MTX, use of leflunomide or not, use of a biological DMARD (bDMARD) (etanercept, adalimumab, golimumab, certolizumab, infliximab, abatacept, rituximab), specific use of tocilizumab, and use of non-steroidal anti-inflammatory drugs (NSAIDs).

### 2.2. FIB-4 Score

The formula for the FIB-4 score is as follows: [age (years) × AST level (U/L)]/[platelet count (10^9^/L) × ALT level (U/L)^1/2^]. A high FIB-4 score was defined as a score >2 if the patient was >60 years or >1.3 otherwise.

### 2.3. Patient and Public Involvement

Patients were not involved in the design of this study; however, patient organizations were involved in its dissemination and in that of the study results to participants and to wider and relevant patient communities.

### 2.4. Ethics Approval and Consent to Participate

The ESPOIR study was conducted with the approval of the Institutional Review Board of Montpellier University Hospital. All patients gave their signed informed consent to participate in the study (ClinicalTrials.gov identifier: NCT03666091).

### 2.5. Statistical Methods

All quantitative variables are described with the median (interquartile range [IQR]), because it offers a robust measure of central tendency that is less sensitive to extreme values. Categorical variables are described with numbers (percentages). The relations between FIB-4 score at baseline and comorbidities were analyzed with a multivariate linear regression model.

For the main objective, we used a linear mixed-effects model with a random effect of patient to account for repeated measures over time. Patient disease activity evolution (DAS28-ESR, modified Sharp score, etc.) was modeled with the FIB-4 score at inclusion and several clinical measurements (number of swollen joints, CRP level, anti-citrullinated protein/peptide antibodies (ACPAs) and rheumatoid factor (RF), and modified Sharp score at inclusion). Time was incorporated as an interaction term to analyze the dynamics of the clinical measure with time. Sensitivity analyses were conducted using various follow-up times, Generalized Additive Mixed Models, regression models with break-points, and quadratic regressions. These sensitivity analyses were performed to assess the robustness of the findings during both short and long follow-up periods and to evaluate the impact of different time dependency modeling approaches.

To assess the effects of treatment on the evolution of the FIB-4 score over time, a multivariate linear mixed-effects model was fitted with the following time-dependent covariates: use of MTX, biologic treatments, NSAIDs, tocilizumab and leflunomide, and sex. Time was incorporated as an interaction term to analyze investigate the temporal dynamics of treatment effects. For all models, sensitivity analyses were performed with and without the interaction of time.

Survival models were produced for MACES, hypercholesterolemia, diabetes, and mortality. For each of these comorbidities, a Cox proportional-hazards model was fitted with adjustment for sex, age, body mass index, and mean FIB-4 score over the first 2 years of follow-up, considering that these successive 2-year measurements at the beginning of RA management were a good reflection of the FIB-4 score throughout the follow-up. Proportional hazard assumption was systematically tested for each covariate, and hazard ratios (HRs) and 95% confidence intervals (CIs) were estimated.

All analyses were performed with R 4.1.0. *p* < 0.05 was considered statistically significant. No alpha risk correction was performed because the study was exploratory. The results should be confirmed in specific clinical studies.

## 3. Results

**Figure 1:**
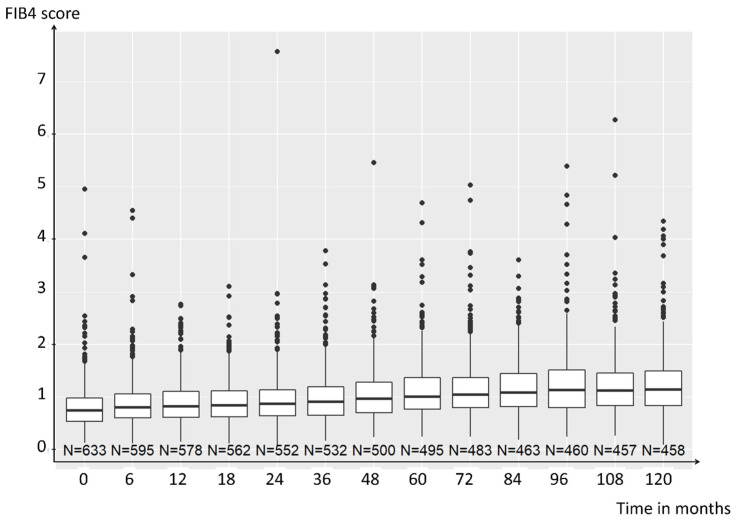
**Fibrosis-4 (FIB-4) score evolution over time.** Data are median (horizontal line), interquartile range (box edges), and range (whiskers).

**Figure A1:**
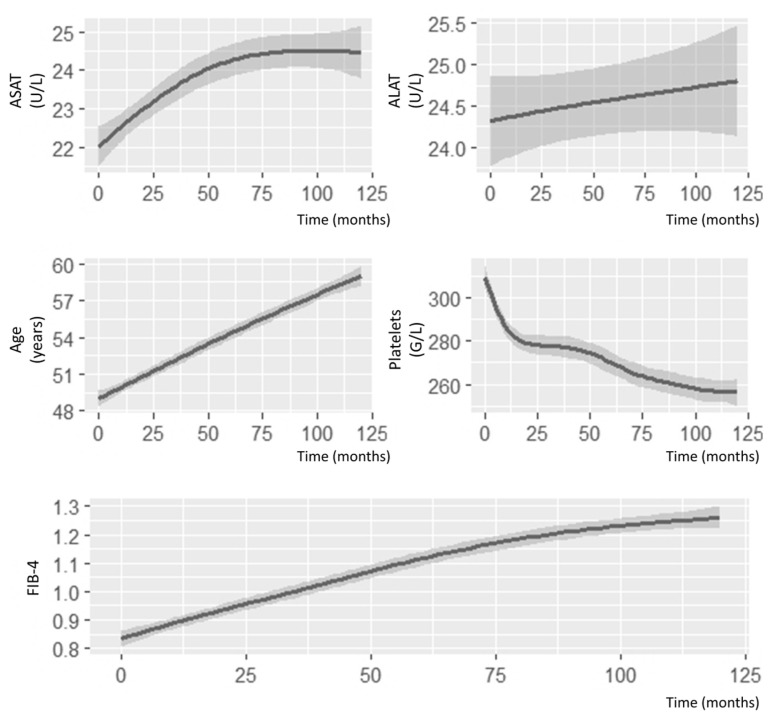
Evolution over time of FIB-4 and its components.

Overall, 647 of the 813 patients included met the ACR/EULAR criteria for RA, with no differential diagnosis during the first 10 years of follow-up. Of these patients, at baseline, 633 had a calculable FIB-4 score. Median FIB-4 score was 0.75 (IQR 0.53–0.99), and 61 (9.6%) patients had a high FIB-4 score at baseline. The main baseline patient characteristics are shown in Table 1. The FIB-4 score evolution over time is shown in Figure 1. FIB-4 tends to increase over time due to the presence of age in its formula. The evolution over time of its various components is shown in Appendix A Figure A1.

### 3.1. FIB-4 Score Association with Comorbidities

**Figure A2:**
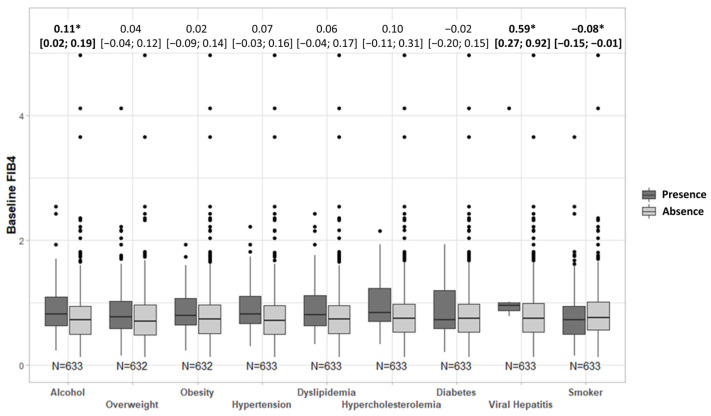
**Baseline FIB-4 by presence or absence of comorbidities.** Data are median (horizontal line), interquartile range (box edges), and range (whiskers). * Statistically significant at *p* < 0.05.

Baseline FIB-4 score was significantly increased for patients with chronic alcohol consumption (mean difference 0.11 [95% CI 0.02; 0.19], *p* = 0.021) or viral hepatitis (0.59 [0.27; 0.92], *p* < 0.001). It was reduced for active smokers (−0.08 [−0.15; −0.01], *p* = 0.018), but we found no other difference concerning other comorbidities (Appendix A Figure A2).

### 3.2. FIB-4 Score Association with Disease Activity and Radiographic Progression

**Figure 2:**
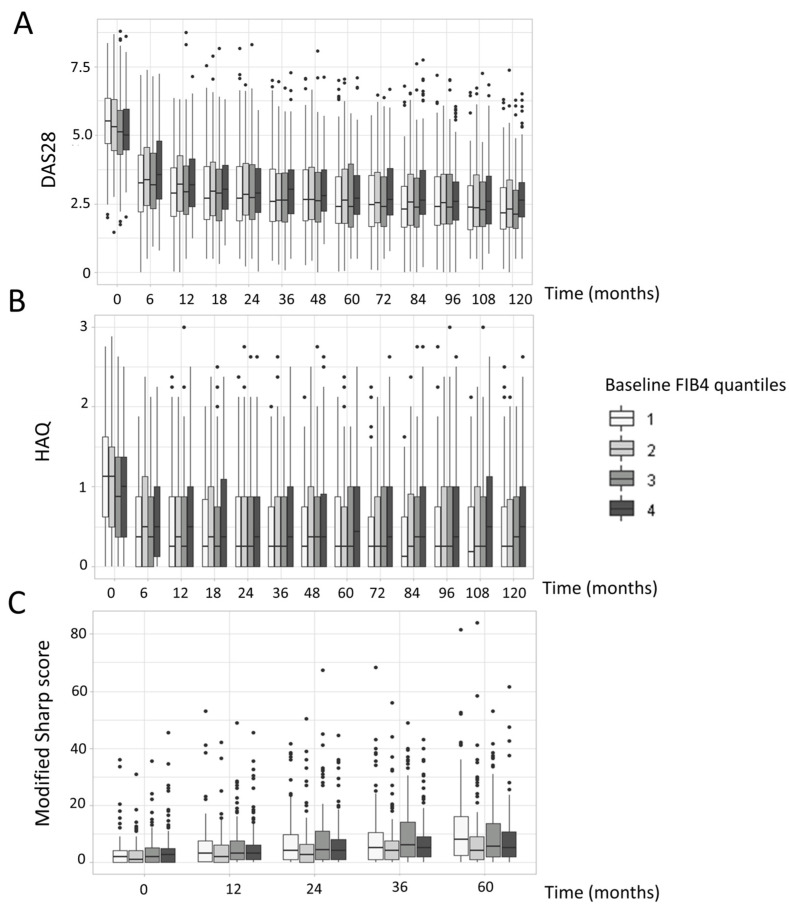
**Impact of baseline FIB-4 score on DAS28 (A), HAQ (B), and total modified Sharp score over time (C).** Data are median (horizontal line), interquartile range (box edges), and range (whiskers). DAS28, Disease Activity Score in 28 joints; HAQ, Health Assessment Questionnaire.

On multivariate analysis including the main known risk factors for unfavorable progression of RA (i.e., number of baseline swollen joints, baseline CRP level, baseline ACPAs and RF, and modified Sharp score), FIB-4 score was not independently associated with evolution of DAS28-ESR over 2 years (Table 2) but was associated with baseline CRP level and number of swollen joints. Baseline FIB-4 score was not associated with the modified Sharp score over 5 years, unlike age and ACPA positivity (Table 2). Different models (Generalized Additive Mixed Models, regression models with break-points, and quadratic regressions) and different follow-up times were tested to try to model RA progression, but none showed a significant clinical link between baseline FIB-4 score and disease progression (Figure 2).

### 3.3. FIB-4 Association with Comorbidities and Mortality

Mean FIB-4 score over the 2 first years of follow-up was not associated with the presence or occurrence of MACEs (HR 0.46 [95% CI 0.13; 1.6], *p* = 0.22), hypercholesterolemia (0.74 [0.42; 1.3], *p* = 0.30), diabetes (1.1 [0.35; 3.2], *p* = 0.92), or death (1.1 [0.46; 2.8], *p* = 0.77) during the 10-year follow-up.

### 3.4. Effect of Treatments on FIB-4 Score

During the 10-year follow-up, 470 patients received MTX, 117 leflunomide, 176 bDMARDs (excluding tocilizimab), 20 tocilizumab, and 484 NSAIDs. We found no significant associations with FIB-4 score evolution in our models including MTX, NSAIDs, tocilizumab, or other bDMARD use (Table 3). Conversely, the use of leflunomide was independently associated with FIB-4 score (mean difference 0.00136, [95% CI 0.000179; 0.00254], *p* = 0.024; Table 3). With all other variables equal, if two patients, one on leflunomide and the other not, have the same FIB-4 score at the visit in month “n”, then in month “n + 1”, the patient on leflunomide would have a FIB-4 score increased by 0.00136 as compared with the one not on leflunomide, which would represent an increase in FIB-4 score of 0.16 after 10 years of follow-up.

## 4. Discussion

The identification of prognostic factors to predict the course of RA or its comorbidities is valuable for the rheumatologist. Here, we aimed to assess the interest of using FIB-4 score, which can be easily assessed during a consultation, as a tool to help to predict the course of RA. To our knowledge, this is the first time this score has been evaluated in a prospective cohort.

The study has notable strengths, including its prospective design over a 10-year period within the ESPOIR cohort, providing valuable insights into the long-term dynamics of RA. The comprehensive data collection enables a thorough examination of demographic, clinical, and treatment-related factors. The use of this early polyarthritis cohort also allows for avoiding the effects of baseline use of DMARDs (because patients were DMARDs-naïve) and to follow the impact of treatments during the 10-year follow-up. Indeed, many RA treatments, such as NSAIDs, MTX, leflunomide, and tocilizumab, are known to affect liver enzyme results. Another strength of our study was to take into account the interaction with time, which we felt was essential given the natural increase in FIB-4 score over time because the FIB-4 score includes age in its formula. This observation might explain in part the discrepancy between the present study, which did not find any association between FIB-4 and mortality, and a previous retrospective study, which did not take into account the interaction with time [12].

The 10-year longitudinal follow-up of ESPOIR also allowed for analyzing the association between FIB-4 score and the evolution of disease activity, radiographic damage, and comorbidities. Our analyses did not find any theranostic role for FIB-4 score. The results of several sensitivity analyses failed to show any clinically meaningful association between the baseline FIB-4 score and the progression of rheumatoid arthritis outcomes. Additionally, no significant difference in disease progression was observed between patients with high and normal FIB-4 profiles at baseline. Despite not revealing a significant association between the FIB-4 score and certain parameters, our in-depth statistical analyses confirmed well-established associations in the literature. Specifically, our study consistently found links with the 2-year evolution of DAS28-ESR (Table 2), as well as with factors such as baseline CRP levels and the number of swollen joints. For the radiographic progression evaluated by the 5-year evolution of the Sharp score, we found a link with ACPA positivity. These findings enhance the internal validity of our study, underlining the consistency of our results with well-established parameters in the context of RA.

Another interest of the present study was to analyze the evolution of the FIB-4 score prospectively in terms of the initiation of DMARDs. Reassuringly, we did not find any significant association between change in FIB-4 score and the use of MTX, NSAIDs, tocilizumab, or other DMARDs, which agrees with a cross-sectional monocentric study [16]. Leflunomide was the only treatment statistically associated with an increase in FIB-4 score over time but without any clinical relevance.

However, several limitations should be acknowledged. Variability in treatment regimens, including changes over the 10-year follow-up, may introduce complexities in interpreting treatment effects. Additionally, the study’s generalizability may be influenced by its focus on a specific cohort, and external factors such as lifestyle and socioeconomic status, which could impact the FIB-4 score, were not comprehensively addressed. Despite these limitations, the study contributes valuable insights into the absence of prognostic value of the FIB-4 score in RA, though further research is warranted to address these constraints and validate the findings across diverse patient populations.

## 5. Conclusions

FIB-4 score is a simple composite score, routinely achievable, but not associated with worse outcome in RA. FIB-4 score was not associated with increased mortality or the occurrence of cardiovascular events. Reassuringly, FIB-4 score was not influenced by treatments, even after 10 years of follow-up.

## Figures and Tables

**Table 1:** Main baseline characteristics of patients (n = 647).

**Age, years, median (IQR)**	**51.0 (40.4** **–** **57.5)**
**Female, n (%)**	500 (77.3%)
**Male, n (%)**	147 (22.7%)
**FIB-4 score** , median (IQR) *	0.75 (0.53–0.99)
High FIB-4 score ** **, n (%)**	61 (9.6%)
**Follow-up in months**	
Mean (±SD)	102.4 (±35.4)
Median (IQR)	120 (108–120)
**Swollen joint count** **, median (IQR**	6 (4–11)
**Tender joint count** **, median (IQR)**	7 (4–13)
**DAS28-ESR** **, median (IQR)**	5.2 (4.5–6.1)
**ESR, mm at 1st hour** **, median (IQR)**	24 (12–39)
**CRP level, mg/l** **, median (IQR)**	10 (5–26)
**Modified total Sharp score,** **median (IQR)**	2 (0–4)
**Rheumatoid factor, IgM,** **median (IQR)**	12 (4–72.5)
**HAQ score,** **median (IQR)**	1.0 (0.5–1.5)
**Positive for rheumatoid factor** **, n (%)**	325 (50.2%)
**Anti-citrullinated protein antibodies** **, median (IQR)**	0 (0–501.5)
**Positive for anti-citrullinated protein antibodies** **, n (%)**	286 (44.6%)
**Weight, kg,** **median (IQR)**	66.3 (58.0–76.0)
**Height, m,** **median (IQR)**	1.64 (1.59–1.70)
**BMI, kg/m^2^,** **median (IQR)**	24.3 (21.9–27.7)
**Overweight (BMI > 25 kg/m^2^), n (%)**	274 (42.3%)
**Obese (BMI > 30 kg/m^2^), n (%)**	89 (13.8%)
**History of myocardial infarction, n (%)**	6 (0.9%)
**History of stroke, n (%)**	4 (0.6%)
**History of major cardiovascular event (MACEs), n (%)**	10 (1.5%)
**History of viral hepatitis**	7 (1.1%)
**Hypertension, n (%)**	117 (18.1%)
**Hypercholesterolemia, n (%)**	98 (15.1%)
**Hypertriglyceridemia, n (%)**	21 (3.2%)
**Triglycerides, mmol/L**	1.0 (0.8–1.5)
**Total cholesterol, mmol/L**	5.2 (4.5–6.0)
**HDL cholesterol, mmol/L**	1.4 (1.2–1.8)
**Smoker, n (%)**	
**Ever**	313 (48.4%)
**Never**	334 (51.6%)
**Current**	145 (22.4%)
**Diabetes, n (%)**	27 (4.2%)
**Chronic alcohol consumption, n (%)**	119 (18.4%)
**Alcohol consumption, if any, g per day,** **median (IQR)**	10 (7–25)

* 647 patients were included in analyses. Data concerning FIB-4 at baseline were missing for 14 patients. ** A high FIB-4 score was defined as a score >2 if the patient was >60 years or >1.3 otherwise. IQR: interquartile range; BMI: body mass index; CRP: C-reactive protein; DAS28-ESR: Disease Activity Score in 28 joints—erythrocyte sedimentation rate; FIB-4: Fibrosis-4; HDL, high-density lipoprotein.

**Table 2:** Multivariate analysis of association of FIB-4 score with DAS28 and modified Sharp score evolution.

Variable	Variables Included in Model	Mean Difference	95% CI	*p*-Value
**DAS28-ESR**	Time	−1.40 × 10^−1^	−0.19; −0.097	<0.0001
	Baseline age	2.00 × 10^−4^	−0.0095; 0.0099	0.97
	Baseline number of swollen joints	1.20 × 10^−1^	0.11; 0.14	<0.0001
	Baseline rheumatoid factor	7.50 × 10^−5^	−1.5 × 10^−4^; 3.0 × 10^−4^	0.51
	Baseline ACPA (presence)	−3.80 × 10^−3^	−0.21; 0.20	0.97
	Baseline CRP	1.10 × 10^−2^	0.0074; 0.014	<0.0001
	Baseline modified Sharp score > 0	1.60 × 10^−1^	−0.056; 0.38	0.15
	Baseline FIB-4	−1.50 × 10^−1^	−0.40; 0.11	0.26
	Baseline age: time	7.10 × 10^−4^	−0.00031; 0.0017	0.17
	Baseline number of swollen joints: time	−9.40 × 10^−3^	−0.011; −0.0075	<0.0001
	Baseline rheumatoid factor: time	1.10 × 10^−6^	−2.2 × 10^−5^; 2.4 × 10^−5^	0.92
	Baseline ACPA (presence): time	8.00 × 10^−3^	−0.014; 0.030	0.47
	Baseline CRP: time	−9.40 × 10^−4^	−0.0013; −0.00060	<0.0001
	Baseline modified Sharp score >0: time	2.10 × 10^−2^	−0.0018; 0.044	0.071
	Baseline FIB-4: time	2.10 × 10^−2^	−0.0053; 0.048	0.21
**Modified Sharp score**	Time	4.30 × 10^−2^	−0.00038; 0.086	0.052
	Baseline age	1.10 × 10^−1^	0.049; 0.17	0.0005
	Baseline number of swollen joints	5.50 × 10^−2^	−0.067; 0.18	0.38
	Baseline rheumatoid factor	−3.80 × 10^−4^	−0.0019; 0.0011	0.61
	Baseline ACPA (presence)	1 . 70	0.38; 3.1	0.012
	Baseline CRP level	2.10 × 10^−3^	−0.017; 0.022	0.84
	Baseline FIB-4	−1.00	−2.7; 0.69	0.25
	Baseline age: time	7.50 × 10^−4^	−0.00017; 0.0017	0.11
	Baseline number of swollen joints: time	3.20 × 10^−4^	−0.0014; 0.0020	0.71
	Baseline rheumatoid factor: time	−4.80 × 10^−6^	−2.4 × 10^−5^; 1.4 × 10^−5^	0.62
	Baseline ACPA (presence): time	5.00 × 10^−2^	0.031; 0.069	<0.0001
	Baseline CRP level: time	6.40 × 10^−4^	0.00035; 0.00093	<0.0001
	Baseline FIB-4: time	−1.90 × 10^−2^	−0.043; 0.0040	0.11

To assess the association of baseline FIB-4 score with the evolution of DAS28 over 2 years and modified Sharp score over 5 years, a multivariate linear mixed-effects model was adjusted on baseline covariates, in interaction with time. Variables without the interaction with time test for the mean differences in the DAS28-ESR/modified Sharp score according to each baseline covariate. Variables with a time interaction test for differences in the evolution of DAS28-ESR/modified Sharp score over time according to the same baseline covariates. For instance, DAS28-ESR is 0.0038 smaller, on average, for patients with ACPAs at baseline. However, between two patients with the same DAS2-ESR at a given time, one with ACPAs at baseline and one without, DAS28 one month later is 0.008 smaller, on average, for the patient with ACPAs. None of these mean differences were statistically significant. CI: confidence interval.

**Table 3:** Multivariate analysis of association of treatments with evolution of FIB-4 score.

Characteristic/Treatment	Mean Difference	95% CI	*p*-Value
Time	0.418	0.33; 0.511	<0.001
Sex	0.121	0.04; 0.206	0.005
Methotrexate	−0.129	−0.19; −0.073	<0.001
Leflunomide	−0.039	−0.12; 0.042	0.344
NSAIDs	−0.001	−0.04; 0.037	0.950
bDMARDs	0.078	0.01; 0.142	0.015
Tocilizumab	−0.105	−0.82; 0.606	0.772
Methotrexate: time	0.080	−0.01; 0.172	0.086
Leflunomide: time	0.163	0.02; 0.305	0.024
NSAIDs: time	−0.058	−0.13; 0.009	0.091
bDMARDs: time	−0.061	−0.16; 0.033	0.204
Tocilizumab: time	0.493	−0.32; 1.307	0.235

To assess the association of each treatment with the evolution of FIB-4 score over time, a multivariate linear mixed-effects model was adjusted on the following covariates: use of methotrexate, biologic treatments, NSAIDs, tocilizumab, and leflunomide, as well as sex. An interaction with time was included for every type of treatment. Coefficients are estimated for 10 years of follow-up, although few patients received the same treatment for that long. Differences in FIB-4 score between patients who did or did not receive each treatment are tested with the term without interaction with time, whereas terms in interaction with time test for differences in the evolution of FIB-4 score over time. For instance, FIB-4 score is smaller by 0.129, on average, in patients who receive methotrexate, which is significantly smaller. However, between two patients with the same FIB-4 score at a given time, one who received methotrexate and one who did not, FIB-4 score one month later is higher by 0.080, on average, for the patient with methotrexate, which is not significantly higher. CI: confidence interval; bDMARDS, biologic disease-modifying antirheumatic drugs; NSAIDs, non-steroidal anti-inflammatory drugs.

## Data Availability

The data that support the findings of this study are available from the ESPOIR cohort committee, upon reasonable request.
